# A Kinematics Analysis Of Three Best 100 M Performances Ever

**DOI:** 10.2478/hukin-2013-0015

**Published:** 2013-03-28

**Authors:** Maćkała Krzysztof, Antti Mero

**Affiliations:** 1 University of School of Physical Education, Department of Track and Field.Wrocław, Poland; 2 University of Jyvaskyla, Department of Biology of Physical Activity. Finland

**Keywords:** sprinting, world record, kinematic analysis, anthropometric characteristics

## Abstract

The purpose of this investigation was to compare and determine the relevance of the morphological characteristics and variability of running speed parameters (stride length and stride frequency) between Usain Bolt’s three best 100 m performances. Based on this, an attempt was made to define which factors determine the performance of Usain Bolt’s sprint and, therefore, distinguish him from other sprinters. We analyzed the previous world record of 9.69 s set in the 2008 Beijing Olympics, the current record of 9.58 s set in the 2009 Berlin World Championships in Athletics and the O lympic record of 9.63 s set in 2012 London Olympics Games by Usain Bolt. The application of VirtualDub Programme allowed the acquisition of basic kinematical variables such as step length and step frequency parameters of 100 m sprint from video footage provided by NBC TV station, BBC TV station. This data was compared with other data available on the web and data published by the Scientific Research Project Office responsible on behalf of IAAF and the German Athletics Association (DVL). The main hypothesis was that the step length is the main factor that determines running speed in the 10 and 20 m sections of the entire 100 m distance. Bolt’s anthropometric advantage (body height, leg length and liner body) is not questionable and it is one of the factors that makes him faster than the rest of the finalists from each three competitions. Additionally, Bolt’s 20 cm longer stride shows benefit in the latter part of the race. Despite these factors, he is probably able to strike the ground more forcefully than rest of sprinters, relative to their body mass, therefore, he might maximize his time on the ground and to exert the same force over this period of time. This ability, combined with longer stride allows him to create very high running speed - over 12 m/s (12.05 – 12.34 m/s) in some 10 m sections of his three 100 m performances. These assumption confirmed the application of Ballerieich’s formula for speed development. In most 10 m sections of the 100 m sprint, the step length was the parameter that significantly determined the increase of maximal running speed, therefore, distinguishing Bolt from the other finalists.

## Introduction

In the last four years Usain Bolt improved the world record in the 100 m sprint three times, from 9.74 s to 9.58 s. Over the last 40 years this record has been revised up to thirteen times from 9.95 s to 9.58 s. The improvement equals 0.37 s (from 1968 to 2009) which is an increase in performance of 3.72%. By comparison, during the same time period, the 200 m world record was revised six times from 19.83 s to 19.19 s what amounts to 3.33 %.

Sprinting speed is defined with the frequency and the length of strides (Mann and Herman, 1985; Ae et al., 1992; Delecluse et al., 1998; Brűggemann et al., 1999; Gajer et al., 1999; Ferro et al., 2001). These parameters are mutually dependant with their optimal ratio enabling maximal sprinting speed. The increase of speed can be achieved by increased length or frequency of strides. The increase of both parameters simultaneously is quite difficult due to mutual dependency. Therefore an increase in one factor will result in an improvement in sprint velocity, as long as the other factor does not undergo a proportionately similar or larger decrease (Hunter at el., 2004). Increased frequency results in shorter stride length and vice versa. Therefore the increase in stride length must be directly proportional with the decrease of stride frequency, especially at the beginning of the race – the initial acceleration phase (Mackala, 2007). This relationship is individually conditioned with the processes of neuro-muscular regulation of movement, morphological characteristics, motor abilities and energy substrates (Mann and Sprague, 1980; Mero et al., 1992; Harland and Steele, 1997; Novacheck, 1998; Coh et al., 2001; Prampero et al., 2005).

According to Hunter et al. (2004) and Bezodias et al. (2008), research investigating the relative importance of developing a long stride length or a high stride rate has been inconsistent across published data. Mann and Herman (1985), Ae et al. (1992) and Bezodias et al. (2008) suggested that SF was a more important contributor to the velocity increase in sprint performance, where Mero and Komi (1985), Gajer et al. (1999), Shen (2000) and Mackala (2007) stated that SL was a more significant variable. However, it is not clear how those two kinematic parameters interact with each other across the entire distance of 100 m in order to accurately identify different phases of the sprint race. No data exist on how world class sprinters manipulate stride frequency and stride length in order to reach optimal efficiency of the sprint run.

The purpose of this investigation was to compare and determine the relevance of the morphological characteristics and variability of running speed parameters (stride length and stride frequency) between Usain Bolt’s three best 100 m performances. Based on this, an attempt was made to define which factors determine the performance of Usain Bolt’s sprint and, therefore, distinguish him from other sprinters.

The presented reasoning leads to the following hypotheses: the stride length is the main factor that determines the increase of running speed in particular 10 m sections of the entire 100 m distance. Knowledge of the relative influence of stride length or stride frequency on maximal running speed would be of great value to coaches and provide a basis for developing specifically designed training protocols for maximum speed development.

## Material and Methods

### Participants

The participants for this study were: a Jamaican sprinter, Usain Bolt (body height = 196 cm, body mass = 93 kg, age = 26 years), current 100 m world record holder (9.58 s) and other world class male sprinters, finalists of: 2008 Beijing Olympic Games (age = 25.3 ± 2.93 years, body height = 176.0 ± 8.40 cm, body mass = 76.7 ± 6.41 kg, and 100 m performance = 9.96 ± 0.05 s (the best result: 9.98 s)), 2009 Berlin World Championships in Athletics (age = 26.7 ± 4.07 years, body height = 177.3 ± 6.40 cm, body mass = 79.0 ± 8.01 kg, and 100 m performance = 9.91 ± 0.10 s (the best result: 9.71 s)), and 2012 London Olympic Games (age = 27.0 ± 3.26 years, body height = 179.4 ± 8.10 cm, body mass = 80.4 ± 8.27 kg, and 100 m performance = 9.86 ± 0.10 s (the best result: 9.75 s)). They were assigned to 2 groups: Usain Bolt and other finalists (n=7 or n=6).

### Anthropometric characteristics

Body mass, body height, body mass index (BMI) and age were collected for each participant from:

- www.2008.NBColympics.com/track/athletes for 2008 Beijing Olympic Games finalists (n=8),
- www.trackandfield.about.com/profiles for 2009 Berlin World Championships in Athletics finalists (n=8), and
- www.BBC.uk/sport/olimpics/2012/athletes for 2012 London Olympic Games finalists (n=8).

The measurements take into account the changes in age and body mass, if there were such for the sprinters taking part in all three finals. Based on measurements of body mass the current BMI was established. BMI was calculated as the ratio of body mass to the squared standing stature (kg·m^−2^).

### Measures and procedures

The data collections were conducted at the 2008 Beijing Olympic Games, 2009 Berlin World Championships in Athletics and 2012 London Olympic Games. The data was obtained from video footage provided by NBC TV stations and BBC TV station and available due to the courtesy of TV on the web. After downloading the video footage from the network in order to see the movie frame by frame, the VirtualDub Programme was applied. The VirtualDub Programme is a video capture/processing utility for 32-bit and 64-bit Windows platforms (98/ME/NT4/2000/XP/Vista/7), licensed under the GNU General Public License (GPL). Through this program, we developed a file format of individual frames where the frames in the video were counted. This enabled the acquisition of basic kinematic data such as: division of the 100 m distance into 10 m sections, average interval time, the number of strides performed, the average stride length calculations, average stride frequency, as well as calculation of running speed for each 10 or 20 m section, at the end of each 10 or 20 m segment. Our data for each 100 m final was compared with other data posted in the web or available by courtesy of the TV station. In turn, our data for the Olympic Games in Beijing in 2008 was compared with data from *speedenduramce.com*. However, this data analyzed only five - 20 m sections. The 100 m final in Berlin in 2009 was compared with data obtained and published by the Scientific Research Project Office responsible on behalf of the IAAF and the German Athletics Association (Berlin 2009) for a research project carried out during the competition. The data regarding 100 m final from 2012 London Olympic Games applies only to Usain Bolt and was published by the Spanish newspaper El Pais. The application of VirtualDub Programme allowed measurement only for a selected sprinter. Accurate measurements from a camera were possible only fore those sprinters not visually obscured or interfered by other sprinters (placement of the foot on the track). This problem occurs at the beginning of the race (acceleration phase), and between 60 and 90 m of the sprint. The material was read and later developed from commercial recording (TV – NBC, BBC) not from the biomechanical set-up on the stadium directly during 100 m performance. This may imply the inaccuracies and missing data. However, data was compared with the sources and no significant differences were found.

### Statistical analysis

In the analysis descriptive statistics were applied. It included the calculation of mean, SD and V (variation). All data were analyzed using the statistics package for windows Statistical Package for Social Science (v. 11,0, Chicago Il.).

## Results

Despite the lack of strong evidence (relationship) between body composition and results in the 100 m sprint, we decided to utilize the information of morphological characteristics of Usain Bolt and compare it with other finalists. This information could not be disregarded especially when analyzing the relationship between running speed, length and frequency of strides and the result in sprinting.

Physically, with body height of 196 cm, Bolt is one of the tallest sprinters in the world. The current second highest sprinter is Ryan Bailey from USA of 193 cm. For comparison, Bolt’s height is almost 20 cm, 18.7 cm and 16.6 cm greater than the average height of the other finalists (2012 Beijing Olympic Games, 2009 Berlin World Championships in Athletics and 2012 London Olympic Games). On the other hand, body mass shows even greater differences between Bolt and the other sprinters. Bolt, despite greater height, is also heavier than the other sprinters: Beijing 14.8%, Berlin 12.3% and 10.7% in London. The disclosed values directly reflect the level of BMI, although the differences are less pronounced (Table 1).

Table 2 contains the basic parameters of three best Usain Bolt sprint performances, compared with the rest of finalists. Bolt completed all three fastest 100m races in an average of 41.13 strides, starting with smaller steps at the beginning of the sprint and covering an average of 2.45 meters with each one. His opponents took about 43–48 strides (the average was: 45.65 strides in Beijing 2008, 44.91 strides in Berlin 2009 and 44.45 strides in London 2012, which gives the average stride length for each competitions (2.19 cm, 2.23 cm and 2.25 cm respectively)). Of course, the stride length is inextricably linked to its frequency, which is about 0.30 Hz lower in Usain Bolt, than in the remaining finalists. Comparing this with Bolt’s times (average of three races – 9,63 s) with that of his rivals (the average of three races - 9.91 s) gives an opportunity to see how he is able to perfectly manage those two kinematic variables in order to reach 12,34 m/s in some 10 m sections of his 100 m performances

Table 3 contains Usain Bolt’s 100 m kinematic data with a breakdown to 10 m sections. This table gives us some real insight into Bolt’s races. It showed that most of the times in each 10 m section from Bejing are similar to those from Berlin and London. The differences are on average of 0.02 seconds. Clear differences can be observed only in the first and last 10 m. Despite the better reaction in comparison to the final in Beijing (a difference of 0.019 s), Bolt’s first 10 m in Berlin was slower by 0.04 s (total time of the first 10 m section was 1.89 s) compared to his race in Beijing. The second significant difference is in the last 10 m. It cost him about 0.07 s compared to the time needed for the last 10 m in Berlin. That is an important time difference for 10 m. After deducting the reaction time (0.019 s) from the first 10 m section (1.85 s) performed in Beijing, we can suppose that Bolt would be able to sprint the first 10 m in 1.83 s, or about 0.06 s faster than he did in the final event in Berlin. Thus, a new world record in the 100 m probably would be 9.52 seconds. In London final from 80 to 100 meters Bolt actually began to slow down. We can see that his time for the last 20 meters is 0.06 seconds slower than his fastest 20 meter split of 1.61 seconds. We also know that he reached his average maximum speed 12.34 s (between 60–80 m).

**Figure 1:**
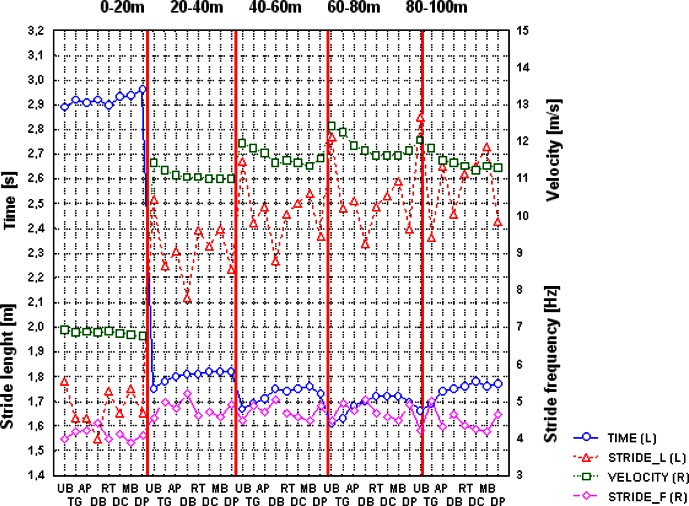
Individual characteristics of groups of selected kinematic parameters from the 100 m final of 2009 Berlin World Championships in Athletics

To better illustrate the differences between Usain Bolt and other world-class sprinters, Table 4 and Figure 1 compared the kinematic fundamental values of the 100 m final in 2009 from Berlin divided into 20 m sections. The individual characteristics of selected kinematic parameters in 100 sprint showed significant differences between sprinters.

Table 5 contains the basic criterion for determining the effectiveness of the speed curve of 100 m sprint. The changes of speed value depend on mutual relations between the stride length and stride frequency. It is evident that up to 40 m at the same time the running speed increases, due to a linear increase in both the stride length and stride frequency. In the later part of the distance, there is a further increase in velocity, although the length and frequency demonstrated a large variable.

## Discussion

The purpose of this investigation was to compare and determine the relevance of the morphological characteristics and variability of running speed parameters (stride length and stride frequency) between Usain Bolt’s three best 100 m performances. Based on this, an attempt was made to define which factors determine the performance of Usain Bolt’s sprint and, therefore, distinguish him from other sprinters.

The influence of Usain Bolt’s biological attributes (body height and body mass) on the stride length and stride frequency would be a simplified explanation for his superiority and elements that significantly distinguish him from rest of the finalists. With regard to the body build, type of muscles (dominated by fast-twitch muscle fibers) and training program, there must be some variables which distinguish Bolt from the rest of current world sprinters. Taking into consideration that in sprinting, there are numerous limiting factors such as gravity, ground contact time, muscle build, power that generates the speed of muscle contractions, it may be assumed that some of these variables make him a super athlete, much faster than other sprinters .

Therefore, it is worth noting that Bolt’s body height and long lower extremities may be also perceived as a disadvantage taking into consideration the fact that he is much slower than his shorter opponents at the acceleration phase. On the other hand, longer lower limbs enable Bolt to propel him in the phase with maximum speed (50–80 m) and allow him to maintain his speed till the end of the race. Bolt’s body height resulting in long strides makes it possible for him to maintain high speed for a longer time and decelerate at a slower rate than shorter sprinters.

On the contrary, fast sprinters are able to achieve higher running speed by striking the ground with greater force and much quicker than slower sprinters do. This is probably another mechanical element that distinguishes Usain Bolt from other world class sprinters. How hard and how quickly do elite sprinters strike the ground? Back to Weyand et al. (2000), at top speed, every sprinter takes around a third of a second to pick their foot up and put it down again. As we know sprinting speed is largely determined by how much force a sprinter can apply to the ground. Generally, they have two possibilities to sprint faster: strike the ground during the contact phase harder or exert the same force over a longer period of time. Considering these two options, we can partly explain why Bolt is superior compared to other sprinters. He is probably able to strike the ground more forcefully than the rest of sprinters, relative to their body mass, therefore, he might maximize his time on the ground. If we consider the option to exert the same force over a longer period of time, it can generate more power so it increases stride length. His forefoot lands probably a little bit further in front of the knee than in other sprinters, although, it does place tremendous stress on the hamstrings muscles. Bolt has an efficient force generation through stride thrust, greater hip flexibility and is able to sprint faster than other sprinters who had faster stride rates. He is biomechanically superior in leg/hip movement then other sprinters. Despite his body height, he is able to manage body strength during the start and initial acceleration phase to create greater force against the ground. It is necessary to understand that visually this type of sprinting technique is common for most top class sprinters. The differences are very small, almost invisible, what creates the impression that Usain Bolt is not only faster, but also he applies a more efficient technique. However, the lack of details with regard to kinetic data concerning these assumptions does not preclude scientifically reasonable considerations that Usain Bolt distinguishes himself from other world class sprinters.

Running velocity is the product of stride length and stride frequency *(v = l x f)* (Luhtanen and Komi, 1978; Mero and Komi, 1985). According to Ballereich (1976), an increase in average velocity (v) and, therefore, a decrease in running time (t) for a given distance, can only result from changes of these two parameters: an increase of stride length (with a decrease of number of strides and their frequency) or inversely, a decrease of stride length (with an increase in stride frequency) (Mackala, 2007). In turn, Delecluse et al. (1998) found a linear relationship between the length of the stride and speed. However their research did not find a significant correlation between stride frequency and speed. On the basis of the Ballereich’s (1976) assumption, improving performance in the sprint events depends on five logical possibilities:

1. *V*+ 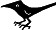
*V* = (*L* + 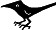
*L*) 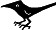
*f* where (*f* ∼ constant)
2. *V*+ 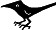
*V* = *L* 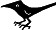 (*f* + 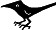
*f)* where (L ∼ constant)
3. *V*+ 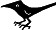
*V* = (*L* + 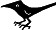
*L*) *x* (*f* + 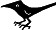
*f*)
4. *V*+ 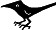
*V* = (*L* + 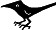
*L*) *x* (*f* - 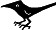
*f*); [(*L* + 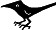
*L*) *x* (*f* - 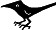
*f*) > *L* 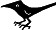
*f*]
5. *V*+ 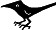
*V* = (*L* - 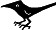
*L*) *x* (*f* + 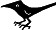
*f*); [(*L* - 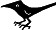
*L*) *x* (*f* + 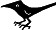
*f*) > *L* 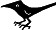
*f*]

Analyzing Table 6, it can be concluded that running speed increased in 21 of 30 analyzed 10 m sections, which represents 70% of their total number. The decline rate was observed in 5 sections (16%), and remaining 4 sections showed constant velocity (13.3%). In half segments (46.7% of all cases), running speed increased (10, 20, 30, 40, 60, 70 m) due to the increase of both kinematic parameters of the sprint V + 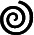
V = (L + 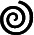
L) x (f + 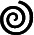
f). With these 7 cases, the length of the stride dominated over the increase of stride frequency, while in 1 case it was the opposite. In 4 cases where the speed was constant compared to previous section, stride length increased three times and stride frequency only once. The decline rate was observed in 5 sections, except for the fact that in three sections, an increase in the length of steps may be noted V - 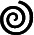
V = (L + 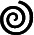
L) x (f- 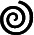
f) and in remaining two sections, an increase occurred in stride frequency V - 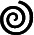
V = (L - 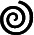
L) x (f + 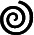
f).

Similar results were presented in early Mackala’s study (2007), which compared the finalists from WC in Tokyo (1991) with the average sprinters. In the final conclusion, the length of stride was a decisive factor with regard to the increase of running speed in each 10 m section. Gajer et al. (1999), who analyzed French best sprinters found that SL was a more important factor contributing to the increase in velocity in sprint performance. Opposite to this statement, Bezodis et al. (2008) conducted a longitudinal case study of stride characteristics in a world class sprinter where the changes in speed occurred as a consequence of changes in stride frequency. This confirms findings of Hunter (2004), who created a map of positive and negative interactions between stride length and stride frequency. He referred to the negative effect that an increase in step length might have on the step rate, and vice versa, as a “negative interaction”. Some authors suggest that stride frequency (Mero et al., 1981; Mero et al., 1992), and related aspects (van Schenau et al., 1994; Wood, 1987) are the speed limiting factors in sprint running; whereas others (Amstrong et al., 1981; Summers, 1997; Shen, 2000; Hunter et al., 2004) indicate that a long stride is more important.

In the present study, the finding confirming the prevalence of stride length over stride frequency was in line with other studies, nevertheless, it should be highlighted that there is a necessity to develop both, a greater stride rate and stride length (Kunz and Kaufman, 1981; Man et al., 1984; Delecluse et al., 1998). The explanation of this phenomenon was discussed by Hunter et al. (2004) who stated that a longer SL is achieved through long term development of strength and power, while SF may be the key factor in developing greater velocity.

According to the authors of this paper, the possibility of a negative or positive interaction between the two discussed kinematic parameters i.e., stride length and stride rate, is rather limited, even though such a statement may be in contradiction with other studies’ results. Furthermore, when considering this issue, it is important to know how an improvement of one factor (i.e., stride length or stride rate) may affect another (Hunter et al., 2000). The main aim consists of reaching an optimal value of both parameters in order to maintain maximal running speed through the entire distance of 100 m. Some results from Table 6 indicate that the sprinter reached optimum relationship (interaction) between stride length and stride frequency. Both variables showed no change in the values;

*Vconst* = (*L* + Δ*L*) · (*f* - *Δf*). This observation should be considered when training an athlete in sprinting.

## Conclusions

Bolt’s anthropometric advantage (body height and lower limbs length) is not questionable and it is one of the factors that makes him faster than the rest of the finalists of each of the three discussed sprinting events. Additionally, Bolt’s almost 20 cm longer stride presents an important benefit in the latter part of the race. Despite these factors, he is probably able to strike the ground more forcefully than other sprinters, relatively to their body mass and, therefore, he might maximize the time of the contact with the ground and apply the same force over this period of time. This ability, combined with longer stride, allows him to reach very high running speed - over 12 m/s (12.05 – 12.34 m/s) in some 10 m sections of his three 100 m performances.

Analysis of the obtained results of this particular sprinter may be of great importance for trainers and coaches as it implies work on stride frequency (SF) in order to reach a higher value of maximal sprinting speed. Therefore, it is noteworthy that the main focus should be on the optimal interaction between stride length and stride frequency.

## Figures and Tables

**Table 1:** Baseline physical characteristics of Usain Bolt and the rest of finalists of the 100 m sprint

Parameters	Olympic Games Beijing 2008 **(1)**			World Championships Berlin 2009 **(2)**			Olympic Games London 2012 **(3)**		
	Usain Bolt	Other Finalists		Usain Bolt	Other Finalists		Usain Bolt	Other Finalists	
		x̄	SD		x̄	SD		x̄	SD
Age (year)	22	25.3	2.93	23	26.7	4.07	26	27.0	3.26
Body mass (kg)	90	76.7	6.41	90	79.0	8.01	93	80.4	8.27
Body height (m)	196	176	3.64	196	177.3	6.40	196	179.4	8.10
BMI (kg /m^2^)	23.4	25.5	2.34	23.4	22.5	2.26	24.2	24.9	1.58

1 data from www.2008.NBColympics.com/track/athletes

2 data from www.trackandfield.about.com/profiles

3 data from www.BBC.uk/sport/olimpics/2012/athletes

**Table 2:** Numerical characteristics of selected kinematic parameters in the 100 m sprint: a) Usain Bolt, b) the rest of finalists

Kinematic parameters **a)** Usain Bolt		Olympic Games Beijing 2008	World Championships Berlin 2009	Olympic Games London 2012	Statistics from three competitions		
					x̄	SD	V
Time [s]		9.69	9.58	9.63	9.63	0.06	0.57
Velocity [m/s]		10.32	10.44	10.38	10.38	0.06	0.58
Stride frequency [Hz]		4.24	4.23	4.29	4.25	0.03	0.76
Number of strides	All	41.1	40.92	41.4	41.13	0.25	0.61
	Take –off from LL	20.7	20.1	20.9	20.57	0.42	2.02
	Take –off from RL	20.4	20.8	20.5	20.57	0.21	1.01
Stride length [m]		2.43	2.47	2.41	2.44	0.03	1.25

Kinematic parameters **b)** the rest of finalists		Olympic Games Beijing 2008 (n=7) **(1, 2, 3)**			World Championships Berlin 2009 (n=7) **(3)**			Olympic Games London 2012 (n=6) **(1, 4, 5)**		
		x̄	SD	V	x̄	SD	V	x̄	SD	V
Time [s]		9.96	0.05	0.51	9.91	0.10	1.06	9.86	0.10	0.93
Velocity [m/s]		10.04	0.05	0.51	10.09	0.11	1.01	10.15	0.09	0.92
Stride frequency [Hz]		4.55	0.21	4.68	4.53	0.20	4.41	4.38	0.19	4.44
Number of strides	All	45.69	2.17	4.75	44.91	1.77	3.94	44.45	1.99	4.48
	Take –off from LL	-	-	-	-	-	-	-	-	-
	Take –off from RL	-	-	-	-	-	-	-	-	-
Stride length [m]		2.19	0.11	4.84	2.23	0.09	3.93	2.25	0.10	4.50

1 data from Waren Doscher;

2 data from adrian.sport.com;

3 data from IAAF –Berlin 2009

4 data from speedendurance.com;

5 hypothetical assumption based on IAAF data

**Table 3:** Selected kinematic parameters (time and velocity) of Usain Bolt in the 100 m sprint: a) Beijing 2008, b) Berlin 2009 and c) London 2012

**a)** Olympic Games Beijing 2008												
Distance [m]	Mean time [s]^*^	mean time [s]^**^	Total time	Mean velocity [m/s]	Total velocity [m/s]	Number of strides	Each 20 m	Total	Stride length [m]	Each 20 m	Stride frequency [Hz]	Each 20 m
0–10	1.85	1.89	1.85	5.40	5.40	7.0	11.1	11.0	1.41	1.82	3.84	3.83
10–20	1.02	0.99	2.87	9.80	6.97	4.1			2.44		4.01	
20–30	0.91	0.90	3.78	10.99	7.94	4.0	8.0	19.0	2.50	2.50	4.39	4.49
30–40	0.87	0.86	4.65	11.49	8.60	3.9			2.56		4.48	
40–50	0.85	0.83	5.50	11.76	9.09	3.9	7.6	26.6	2.56	2.63	4.59	4.55
50–60	0.82	0.82	6.32	12.19	9.49	3.7			2.70		4.51	
60–70	0.82	0.81	7.14	12.19	9.80	3.8	7.4	34.0	2.63	2.70	4.63	4.51
70–80	0.82	0.82	7.96	12.19	10.05	3.6			2.77		4.39	
80–90	0.83	0.83	8.79	12.05	10.24	3.5	7.1	41.1	2.86	2.83	4.22	4.10
90–100	0.90	0.83	9.69	11.11	10.31	3.6			2.77		4.00	

* Data from SportEndurance.com

** Data from VirtualDub

**Table:** 

**b)** World Championship - Berlin 2009												
Distance [m]	Mean time [s]^*^	Mean time [s]^**^	Total time	Mean velocity [m/s]	Total velocity [m/s]	Number of strides	20 m Section	Total	Stride length [m]	Each 20 m	Stride frequency [Hz]	Each 20 m
0–10	1.89	1.90	1.90	5.26	5.26	7	11.2	11.2	1.43	1.78	3.68	3.89
10–20	0.99	0.98	2.88	10.20	6.94	4.2			2.38		4.28	
20–30	0.90	0.92	3.80	10.87	7.90	4.0	7.9	19.1	2.50	2.52	4.35	4.54
30–40	0.86	0.83	4.63	12.05	8.64	3.9			2.56		4.70	
40–50	0.83	0.84	5.46	11.90	9.16	3.8	7.5	26.6	2.63	2.67	4.64	4.49
50–60	0.82	0.82	6.29	12.19	9.53	3.8			2.63		4.63	
60–70	0.81	0.82	7.11	12.19	9.84	3.7	7.2	33.8	2.70	2.77	4.51	4.49
70–80	0.82	0.81	7.92	12.34	10.10	3.5			2.86		4.32	
80–90	0.83	0.83	8.75	12.05	10.28	3.4	7.0	40.8	2.94	2.85	4.10	4.23
90–100	0.83	0.83	9.58	12.05	10.44	3.6			2.77		4.34	

* IAAF official data

** Data from VirtualDub

**Table:** 

**c)** Olympic Games - London 2012												
Distance [m]	Average time [s]^*^	Total time [s]	Total time [s]^**^	Average velocity [m/s]	Total velocity [m/s]	Number of strides	20 m Section	Total	Stride length [m]	Each 20 m	Stride frequency [Hz]	Each 20 m
0–10	1.91	1.91		5.23	5.23	7.3	11.5	11.5	1.37	1.74	3.82	3.94
10–20	1.01	2.92	2.93	9.90	6.84	4.2			2.38		4.16	
20–30	0.92	3.84		10.87	7.81	4.1	8.0	19.5	2.44	2.50	4.45	4.49
30–40	0.86	4.70	4.69	11.63	8.51	3.9			2.56		4.54	
40–50	0.84	5.54		11.90	9.02	4.0	7.8	27.3	2.50	2.56	4.76	4.70
50–60	0.82	6.36	6.35	12.19	9.43	3.8			2.63		4.63	
60–70	0.81	7.17		12.34	9.76	3.6	7.0	34.3	2.78	2.86	4.44	4.32
70–80	0.81	7.98	7.96	12.34	10.02	3.4			2.94		4.20	
80–90	0.82	8.80		12.19	10.23	3.6	7.1	41.4	2.78	2.82	4.38	4.30
90–100	0.83	9.63	9.63	12.05	10.38	3.5			2.86		4.21	

* Data from VirtualDub

** Data from El Pais

**Table 4:** Comparison of the 20 m sections time interval, speed, stride length, stride frequency and stride numbers between Usain Bolt and the rest of athletes of the 100 m final of World Championship – Berlin 2009

Usain Bolt - World Championship - Berlin 2009						The Rest of Finalists - World Championship - Berlin 2009									
Distance (m)	Total Time (s)	Mean velocity [m/s]	Mean stride numbers	Mean stride length (m)	Mean stride frequency (Hz)	Total time (s)		Mean velocity [m/s]		Mean stride numbers		Mean stride length (m)		Mean stride frequency (Hz)	
						x̄	SD	x̄	SD	x̄	SD	x̄	SD	x̄	SD
0–20	2.88	6.94	11.2	1.78	3.89	2.93	0.02	6.83	0.05	12.09	0.51	1.66	0.07	4.13	0.18
20–40	1.75	11.42	7.9	2.52	4.54	1.81	0.01	11.06	0.09	8.73	0.37	2.29	0.10	4.84	0.23
40–60	1.66	12.05	7.5	2.67	4.49	1.73	0.02	11.54	0.17	8.21	0.31	2.44	0.09	4.75	0.20
60–80	1.63	12.26	7.2	2.77	4.49	1.70	0.03	11.80	0.23	8.06	0.27	2.48	0.08	4.77	0.20
80–100	1.66	12.05	7.0	2.85	4.23	1.75	0.03	11.43	0.20	7.81	0.45	2.56	0.14	4.49	0.30

**Table 5:** Differences in interval speed, stride length, stride frequency of Usain Bolt (World Record) in: a) Olympic Games in Beijing 2008, b) World Championships in Berlin 2009 and c) Olympic Games in London 2012

Descriptive statistic	**a)** Olympic Games Beijing 2008									
	10	20	30	40	50	60	70	80	90	100
Absolute change in speed (m/s)	5.40	(+) 4.40	(+) 1.19	(+) 0.50	(+) 0.27	(+) 0.43	(+) 0.00	(+) 0.00	**(-) 0.14**	**(-) 0.94**
Relative change in speed (%)	0.0	81.5	12.1	4.5	2.3	3.6	0.0	0.0	1.2	7.8
Absolute change in stride length (m)	1.41	(+) 1.03	(+) 0.06	(+) 0.06	(+) 0.00	(+) 0.14	(-) 0.07	(+) 0.14	(+) 0.09	(-) 0.09
Relative change in stride length (%)	0.0	73.0	2.45	2.40	0.0	5.5	2.6	5.3	3.2	3.2
Absolute change in stride frequency (Hz)	3.84	(+) 0.17	(+) 0.38	(+) 0.09	(+) 0.11	**(-) 0.08**	(+) 0.12	**(-) 0.24**	**(-) 0.17**	**(-) 0.22**
Relative change in stride frequency (%)	0.0	4.4	9.5	2.0	2.4	1.7	2.7	5.2	3.9	5.2

(+) increase,

(-) decrease,

* Data from VirtualDub

**Table:** 

Descriptive statistic	**b)** World Championship - Berlin 2009									
	10	20	30	40	50	60	70	80	90	100
Absolute change in speed (m/s)	5.26	(+) 4.94	(+) 0.57	(+) 1.18	**(-) 0.15**	(+) 0.29	(+) 0.00	(+) 0.15	**(-) 0.29**	(+) 0.00
Relative change in speed (%)	0.0	93.9	6.5	10.8	1.3	2.4	0.0	1.2	2.4	0.0
Absolute change in stride length (m)	1.43	(+) 0.95	(+) 0.12	(+) 0.06	(+) 0.13	(+0) 0.00	(+) 0.07	(+) 0.16	(+) 0.08	**(-) 0.17**
Relative change in stride length (%)	0.0	66.4	5.0	2.4	2.7	0.0	2.6	5.9	2.8	**5.8**
Absolute change in stride frequency (Hz)	3.68	(+) 0.60	(+) 0.07	(+) 0.35	**(-) 0.06**	(+) 0.01	**(-) 0.12**	**(-) 0.19**	**(-) 0.22**	(+) 0.24
Relative change in stride frequency (%)	0.0	16.3	1.6	8.0	1.3	0.01	3.6	4.2	5.1	5.8

(+) increase,

(-) decrease,

* Data from VirtualDub

**Table:** 

Descriptive statistic	**c)** Olympic Games - London 2012									
	10	20	30	40	50	60	70	80	90	100
Absolute change in speed (m/s)	5.23	(+) 4.67	(+) 0.97	(+) 0.76	(+) 0.27	(+) 0.29	(+) 0.15	(=) 0.00	**(-) 0.15**	**(-) 0.14**
Relative change in speed (%)	0.0	89.3	9.8	6.9	2.3	2.4	1.2	0.0	**1.2**	**1.2**
Absolute change in stride length (m)	1.37	(+) 1.01	(+) 0.06	(+) 0.12	**(-) 0.06**	(+) 0.13	(+) 0.15	(+) 0.16	**(-) 0.16**	**(+) 0.08**
Relative change in stride length (%)	0.0	73.7	5.7	4.9	2.4	5.2	5.7	5.7	5.5	2.9
Absolute change in stride frequency (Hz)	3.82	(+) 0.34	(+) 0.29	(+) 0.09	(+) 0.22	**(-) 0.13**	**(-) 0.19**	**(-) 0.24**	(+) 0.18	**(-) 0.17**
Relative change in stride frequency (%)	0.0	8.9	7.0	2.0	4.8	2.7	4.1	5.4	4.3	4.9

(+) increase,

(-) decrease,

* Data from VirtualDub

**Table 6:** Comparison of the velocity curve for selected phases of the 100 m sprint due to individual changes of the stride rate and stride length

Phase	Distance (m)	Olympic Games Beijing 2008	World Championship Berlin 2009	Olympic Games Berlin 2012
1	0–10	*V +* 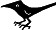 *V = (L + L) x (f +* 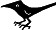 *f)*	*V +* 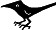 *V* = (*L* + 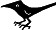 *L*) *x* (*f* + 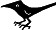 *f)*	*V +* 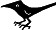 *V* = (*L* + 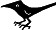 *L*) *x* (*f* + 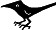 *f)*
2	10–20	*V +* 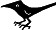 *V* = (*L* + 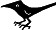 *L*) *x* (*f* + 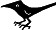 *f)*	*V +* 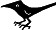 *V* = (*L* + 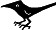 *L*) *x* (*f* + 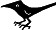 *f)*	*V +* 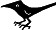 *V* = (*L* + 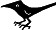 *L*) *x* (*f* + 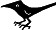 *f)*
3	20–30	*V +* 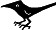 *V* = (*L* + 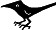 *L*) *x* (*f* + 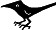 *f)*	*V +* 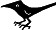 *V* = (*L* + 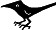 *L*) *x* (*f* + 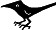 *f)*	*V +* 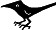 *V* = (*L* + 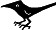 *L*) *x* (*f* + 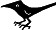 *f)*
4	30–40	*V +* 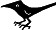 *V* = (*L* + 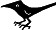 *L*) *x* (*f* + 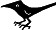 *f)*	*V +* 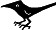 *V* = (*L* + 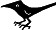 *L*) *x* (*f* + 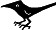 *f)*	*V +* 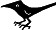 *V* = (*L* + 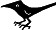 *L*) *x* (*f* + 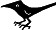 *f)*
5	40–50	*V +* 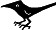 *V* = (*L* + 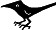 *L*) *x* (*f* + 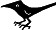 *f)*	*V -* 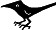 *V* = (*L* + 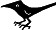 *L) x (f -* 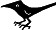 *f)*	*V +* 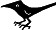 *V = (L -* 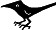 *L) x (f -+* 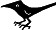 *f)*
6	50–60	*V +* 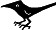 *V* = (*L* + 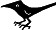 *L) x (f -* 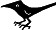 *f)*	*V +* 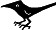 *V* = (*L* + 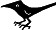 *L*) *x* (*f* + 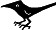 *f)*	*V +* 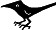 *V* = (*L* + 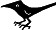 *L) x (f -* 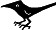 *f)*
7	60–70	V=const *= (L -* 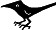 *L*) *x* (*f* + 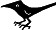 *f)*	*V +* 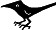 *V* = (*L* + 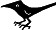 *L) x (f -* 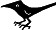 *f)*	*V +* 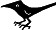 *V* = (*L* + 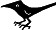 *L) x (f -* 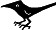 *f)*
8	70–80	V=const *= (L +* 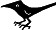 *L) x (f -* 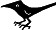 *f)*	*V +* 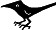 *V* = (*L* + 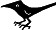 *L) x (f -* 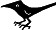 *f)*	V=const *= (L +* 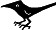 *L) x (f -* 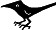 *f)*
9	80–90	*V -* 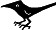 *V* = (*L* + 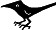 *L) x (f -* 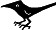 *f)*	*V +* 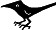 *V* = (*L* + 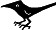 *L) x (f -* 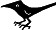 *f)*	*V -* 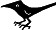 *V = (L -* 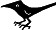 *L*) *x* (*f* + 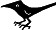 *f)*
10	90–100	*V -* 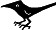 *V = (L -* 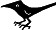 *L) x (f -* 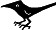 *f)*	V=const *(L -* 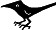 *L*) *x* (*f* + 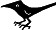 *f)*	*V -* 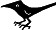 *V* = (*L* + 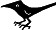 *L) x (f -* 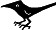 *f)*
